# Influences on the Functional Behavior of Great Arteries during Orthostasis

**DOI:** 10.5935/abc.20190182

**Published:** 2019-12

**Authors:** Jorge Elias Neto, Albano Ferreira, Guilherme Futuro, Luiz Carlos dos Santos, Nevelton Heringer Filho, Fernando Gomes, Jose Geraldo Mill

**Affiliations:** 1 Vitória Apart Hospital, Serra, ES - Brazil; 2 Universidade Katyavala Bwila Benguela, Luanda - Angola; 3 Universidade Federal do Espirito Santo, Vitória, ES - Brazil

**Keywords:** Switch Arterial, Hypertension, Aging, Standing Position, Pulse Wave Analysis, Gravitation

## Abstract

**Background:**

Arterial compliance reduction has been associated with aging and hypertension in supine position. However, the dynamic effects of orthostatism on aortic distensibility has not been defined.

**Objective:**

We sought to determine the orthostatic influence and the interference of age, blood pressure (BP) and heart rate (HR) on the great arteries during gravitational stress.

**Methods:**

Ninety-three healthy volunteers (age 42 ± 16 years). Carotid-femoral pulse wave velocity (PWV) assumed as aortic stiffness was assessed in supine position (basal phase), during tilt test (TT) (orthostatic phase) and after return to supine position (recovery phase). Simultaneously with PWV acquisition, measures of BP and HR rate were recorded.

**Results:**

PWV during TT increased significantly compared to the basal and recovery phases (11.7 ± 2.5 m/s vs. 10.1 ± 2.3 m/s and 9.5 ± 2.0 m/s). Systolic BP (r = 0.55, r = 0.46 and r = 0.39) and age (r = 0.59, r = 0.63 and r = 0.39) correlated with PWV in all phases. The significance level for all tests was established as α = 0.05.

**Conclusion:**

We conclude that there is a permanent increase in PWV during orthostatic position that was returned to basal level at the recovery phase. This dynamic pattern of PWV response, during postural changes, can be explained by an increase in hydrostatic pressure at the level of abdominal aorta which with smaller radius and an increased elastic modulus, propagates the pulse in a faster way. Considering that it could increase central pulse reflection during the orthostatic position, we speculate that this mechanism may play a role in the overall adaptation of humans to gravitational stress.

## Introduction

Great arteries are not only seen as mere passive conductors of blood, functioning only in its transportation and distribution, but rather playing a fundamental and complex role in the maintenance of circulatory homeostasis and in the genesis of cardiovascular disease.^1-2^ The great arteries may be considered a functional organ with several roles, such as endocrine and paracrine activity, in addition to the capacity for muffling the pulsatile blood flow.

The functional behavior of the great arteries in the supine position was assessed noninvasively by measuring the pulse wave velocity (PWV) in several arterial segments.^3-5^ Epidemiological and longitudinal studies using that methodology have shown the clinical relevance of this approach for predicting morbid cardiovascular events.^1,6-7^

However, due to methodological limitations, the functional response of the great arteries has not been investigated in the orthostatic position.^8^

The tilt test has long been used for assessing the influence of gravitational stress on the behavior of hemodynamic parameters.^9-10^ Although this technique allows adequate reproducibility of the gravitational action upon individuals in active orthostatic position, only from the end of the 1980s that small studies have been carried out with primates, aiming at assessing the influence of postural changes on the behavior of aortic pulse wave and PWV.^8,11-13^ Studying the function of the great arteries in the orthostatic position by noninvasively measuring carotid-femoral PWV may be important for understanding the vascular mechanisms of adaptation to gravity and their implications on cardiocirculatory homeostasis, development or progression of cardiovascular disease and occurrence of unadaptable postural events.

This study is the first to assess the effects of orthostatic position on the function of the great arteries in humans, by measuring the carotid-femoral PWV in healthy individuals and in individuals with untreated mild-to-moderate arterial hypertension. We tested the hypothesis that orthostatism could lead to increased PWV compared to the supine positions and considering the influence of blood pressure, age and heart rate.

## Methods

### Patient characteristics

To define the sample size, we used studies that evaluated PWV in supine position.^4-7^

The study included 93 individuals, 74 males and 19 females whose ages ranged from 18 to 75 years (42 ± 16 years). Twenty-nine (31.1%) individuals had systolic blood pressure levels ≥ 140 and/or diastolic blood pressure levels > 90 mm Hg. These individuals either had undiagnosed arterial hypertension or had voluntarily interrupted the antihypertensive treatment for more than 30 days. Their anthropometric and hemodynamic characteristics are shown in Table 1.

**Table 1:** Anthropomorphic and hemodynamic characteristics of the participants

Characteristics		Participants (n = 93)		
Sex		Male (n = 74)		Female (n = 19)
		Mean ± Standard deviation		
Age, years		42	±	16
Weight, kg		71	±	12
Height, cm		1,7	±	0,1
BMI, kg/m^2^		24,7	±	3,1
SBP, mmHg		130	±	18
DBP, mmHg		82	±	13
MBP, mmHg		99	±	15
HR, bpm		66	±	11
PP, mmHg		47	±	13

BMI: body mass index; SBP: systolic blood pressure; DBP: diastolic blood pressure; MBP: medium blood pressure; PP: pulse pressure; HR: heart rate. The continuous values are expressed as mean ± SD.

The exclusion criteria were as follows: clinical history or evidence of any type of cardiac structural disease; overweight or obesity; diabetes mellitus; smoking; dyslipidemia; peripheral vascular disease; chronic renal failure; clinical data suggestive of dysautonomia; and orthostatic intolerance or previous vasovagal events. Patients with arterial hypertension on antihypertensive treatment and patients on any medication that could interfere with the results of the parameters assessed or that could account for the occurrence of orthostatic hypotension during the tilt test were also excluded from the study.

### Automatic measurement of carotid-femoral PWV

Carotid-femoral PWV index, a measure of aortic stiffness, was assessed with an automatic device (Complior, Colson, France) that measures the time delay between the rapid upstroke of the feet of simultaneously recorded pulse waves in the carotid and femoral arteries by using 2 pressure transducers (TY-306 type; Fukuda Deshi Co., Tokyo, Japan). PWV was calculated as the ratio between the distance and the foot-to-foot time delay and was expressed in meters per second. (6). All PWV measurements in the supine position were taken and assessed by a single observer. Obtainment of PWV during the tilt test required 2 researchers, who were acquainted with the technique in the supine position and were trained for PWV measurement in the orthostatic position.

### Protocol of the tilt test associated with PWV measurement

All individuals were assessed in the morning. They were instructed to fast for 12 hours. Prior to the examination, anthropometric measurements of weight, height, and waist and hip perimeters were taken. Then, the individuals were placed in the supine position on a mechanical table of tilt test.

After a 20-minute rest, during which the individuals were instructed about the dynamic sequence of the protocol, the following baseline measurements were taken: PWV, heart rate (HR), systolic blood pressure (SBP), diastolic blood pressure (DBP), mean blood pressure (MBP) and pulse pressure (PP). After that, the patients were tilted at an angle of 70º. The tilt test lasted 20 minutes. The measurements of parameters monitored during tilt testing were performed at 2-minute intervals and 2 minutes after returning to the supine position. Electrocardiographic monitoring was continuously performed. Blood pressure was noninvasively measured at 2-minute intervals using the Omega 1400 monitor (Invivo Research Laboratories, USA) while PWV measurements were being taken or when the patient reported any symptom or had clinical signs or electrocardiographic abnormalities suggestive of that diagnosis.

### Statistical analysis

Anthropometric, biological and hemodynamic characteristics were expressed as mean ± standard deviation (SD). One-way analysis of variance (ANOVA) was used to compare hemodynamic parameters obtained at baseline condition, during the tilt test at 70º (0-2 min, 10 min and 20 min), and in the recovery phase (0-2 min). The correlation between PWV obtained during the protocol and all parameters was determined using Pearson’s correlation coefficient in the entire sample. Then, multiple linear regression was used to assess the influence of the different hemodynamic parameters on the PWV during the tree phase. All assumptions required for regression analysis were verified.

The partial linear correlation coefficients between PWV and age controlled for the blood pressure effect were calculated for each phase of the protocol. Similarly, the influence of age on the correlation of PWV and blood pressure was determined. Then, multivariate analysis of variance (MANOVA) and analysis of covariance (ANCOVA) were performed to assess the independent effects of age and SBP on PWV in the different phases of the protocol. Considering that the 2 major modulating factors of PWV level are age and SBP, the clinical results were assessed after adjustment for these 2 variables. Aiming at assessing the variables that interfere with heart rate behavior in orthostatic position, multiple linear regression was performed. A normality assessment of the data was performed for all variables.

The significance level for all tests was established as α = 0.05. The statistical analyses were performed using the SPSS for Windows software (version 18.0, SPSS Inc., 2010).

## Results

### Effects of the tilt test at 70 on the hemodynamic characteristics of the participants

**Figure 1:**
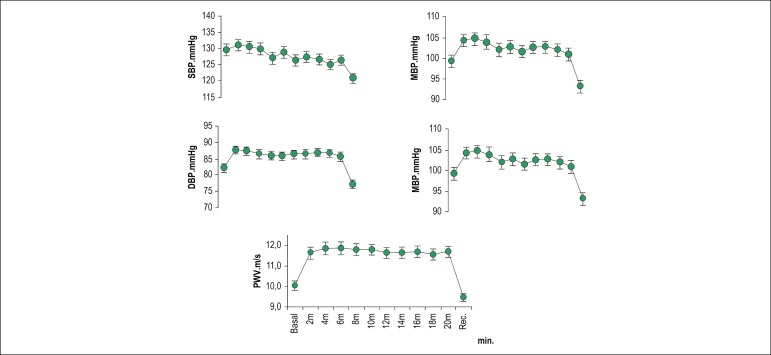
Chart showing the dynamic behavior of hemodynamic parameters monitored during the protocol. 151x155 mm (96 x 96 DPI)

Figure 1 shows the dynamic behavior of the hemodynamic variables during the protocol.

Table 2 shows the mean values of the variables studied at baseline, during the tilt test, and in the recovery phase.

**Table 2:** Effects of the tilt test at 70º/20 min. (mean in standing position) on the participants’ hemodynamic parameters and PWV

Variable	Basal	Tilt test (mean in standing position)	Recovery
	Mean ± SD	Mean ± SD	Mean ± SD
SBP, mmHg	130 ± 1811	128 ± 15	121 ± 16^†††^
DBP, mmHg	082 ± 13***	087 ± 11	077 ± 13^†††^
MBP, mmHg	099 ± 15***	103 ± 11	093 ± 14^†††^
PP, mmHg	047 ± 13***	042 ± 8	044 ± 11^†††^
HR, bpm	066 ± 11***	079 ± 14	068 ± 13^†††^
PWV, m/s	010 ± 2.3***	011 ± 2.5	009 ± 2,0^†††^

*** p < 0.001 vs. tilt test;

††† p < 0.001 vs. tilt test.

SBP: systolic blood pressure; DBP: diastolic blood pressure; MBP: medium blood pressure; PP: pulse pressure; HR: heart rate; PWV: pulse wave velocity.

Based on the analysis of these data, the response of the individuals to postural changes was associated with a 6% increase in DBP, which was maintained during the entire phase of orthostatic position. The same was observed for the MBP value in orthostatic position. On the other hand, PP in orthostatic position decreased by 10.6%. An 11.5%-increase in PWV in orthostatic position (PWVp) was observed as compared with the baseline PWV value.

### Association between PWV in the supine position and anthropometric and hemodynamic characteristics of the participants

The results of the linear correlation analysis between carotid-femoral PWV in the supine position and the anthropometric and hemodynamic parameters are shown in Table 3.

**Table 3:** Correlation between carotid-femoral pulse wave velocity in the supine position and anthropomorphic and hemodynamic parameters

Parameters	Correlation coefficient	p value
Age, years	0.593	< 0.001
Weight, kg	0.063	NS
Height, cm	–0.125	NS
BMI, Kg/m^2^	0.194	NS
SBP, mmHg	0.547	< 0.001
DBP, mmHg	0.528	< 0.001
MBP, mmHg	0.560	< 0.001
HR, mmHg	0.063	NS
PP, mmHg	0.216	< 0.05

BMI: body mass index; SBP: systolic blood pressure; DBP: diastolic blood pressure; MBP: medium blood pressure; HR: heart rate; PP: pulse pressure; NS: no significance.

Multiple linear regression showed that age (p < 0.001) and SBP (p < 0.001) were the only independent predictive variables of baseline PWV. These 2 factors accounted for approximately 50% of the variability observed in baseline PWV (r^2^ = 0.505, p < 0.001).

### Association between mean PWV during the tilt test at 70º and the anthropometric and hemodynamic characteristics of the participants

**Figure 2:**
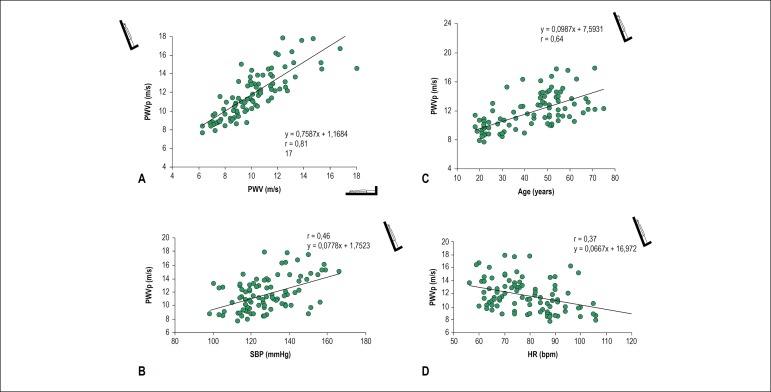
Chart of dispersion between carotid-femoral pulse wave velocity in the standing position (PWVp) and: A – basal carotid-femoral pulse wave velocity (basal PWV), p < 0.01; B – systolic blood pressure (SBP), p < 0.001; C – age, in 93 participants, p < 0.001; D – heart rate (HR) in 93 participants, p < 0.001.

Analysis of the correlation between PWV measurements taken in the supine position (baseline PWV) and PWV during the tilt test (PWVp) showed a significant influence of baseline PWV on the response obtained in orthostatic position (Figure 2A).

Results of the linear correlation analysis between carotid-femoral PWVp obtained during the tilt test and the anthropometric and hemodynamic parameters of the study participants are shown in Table 4.

**Table 4:** Correlation between mean carotid-femoral PWV during the tilt test at 70º/20 min with anthropomorphic and hemodynamic parameters

Parameters	Correlation coefficient	p value
Age, years	0.638	< 0.001
Weight, kg	0.220	< 0.05
Height, cm	–0.020	NS
BMI, kg/m^2^	0.323	< 0.01
SBPp, mmHg	0.464	< 0.001
DBPp, mmHg	0.363	< 0.001
MBPp, mmHg	0.358	< 0.001
HRp, mmHg	–0.366	< 0.001
PPp, mmHg	0.307	< 0.01

BMI: body mass index; SBPp: mean systolic blood pressure during the tilt test; DBPp: mean diastolic blood pressure during the tilt test; MBPp: mean medium blood pressure during the tilt test; HRp: mean heart rate during the tilt test; PPp: mean pulse pressure during the tilt test; NS: no significance.

Once again, multiple linear regression defined age and SBP as the major independent predictive factors of PWV obtained during the tilt test, accounting together for 49% of the variations in PWV during that test (r^2^ = 0.490).

### Association between PWV during the recovery phase and the anthropometric and hemodynamic characteristics of the participants

The correlation of variables obtained when the individuals returned to the supine position 20 minutes after the tilt test (recovery phase) was assessed. This final phase of the study represents an immediate response (0-2 min) of the hemodynamic parameters and their correlation with PWV during the recovery phase (PWVrec). A significant correlation was observed between PWVrec and most variables assessed in this phase of the protocol. Once again, the variable age had the greatest positive correlation (r = 0.533, p < 0.001) with PWVrec. A positive correlation was also observed between PWVrec and the following parameters: BMI (r = 0.26, p < 0.05); SBP (r = 0.39, p < 0.001); DBP (r = 0.49, p < 0.001); and MBP (r = 0.457, p < 0.001). In the recovery phase, a negative correlation between HR (r = 0.055, NS) and PWVrec was no longer observed. As occurred in the 2 preceding phases, multiple linear regression in the recovery phase showed that SBP (p < 0.001) and age (p < 0.001) were independent predictors of variations in PWVrec. Together, these variables accounted for approximately 40% of the variations in PWVrec.

### Analysis of the effects of distensibility pressure on aortic stiffness in the baseline supine position, orthostatic position and after the tilt test

Variation in the carotid-femoral PWV, as an index of aortic stiffness, was assessed with regard to the SBP values, aiming at comparing the effects of distensibility pressure on the mechanical properties of the great arteries in the population (Figure 2B).

The analysis of SBP in the passive orthostatic position showed that SBP accounted for approximately 21% (r^2^ = 0.214, p < 0001) of the variations observed in PWV. After adjusting SBP for age, SBP proved to play an even more significant role in the PWVp behavior pattern (r^2^ = 0.38, p < 0.001).

Finally, in the statistical analysis performed in the recovery phase, SBP continued to play a significant role in the PWVrec variations (r^2^ = 0.15, p < 0.001). The same was observed for SBP adjusted for age (r^2^ = 0.23, p < 0.001).

### Analysis of the effect of aging on aortic stiffness in the baseline supine position, orthostatic position and after the tilt test

To assess the effects of aging on arterial stiffness, the correlation curve (Figure 2C) was performed with regard to age. In all phases of the protocol, PWV significantly increased with age (r^2^ = 0.351, p < 0.001; r^2^ = 0.4066, p < 0.001; r^2^ = 0.283, p < 0.001).

### Assessment of the effect of heart rate on PWV measured in the different phases of the protocol

The influence of heart rate behavior on arterial distensibility was assessed. As previously reported, no correlation was observed between HR and PWV assessed in the baseline and recovery phases.

On the other hand, during the tilt test, a negative correlation was observed between PWVp and HR (Figure 2D).

Heart rate accounted for approximately 13% of the variation in PWV during the tilt test (r^2^ = 0.136, p < 0.001), and this influence continued to be observed even after PWV was adjusted for age (r^2^ = 0.154, p < 0.001). The heart rate decrease in orthostatic position was significantly conditioned to age increase in the participants (r^2^ = 0.27, p < 0.001).

Finally, the correlation between the HR behavior in orthostatic position and the hemodynamic parameters at baseline was assessed. A negative correlation with baseline PWV measurement was observed (r = -0.30, p < 0.01).

## Discussion

The main new finding of this study was the instant and significant increase in PWV in orthostatic position (Table 3). This pattern of vascular functional behavior was present in all individuals studied regardless of age, resulting in PWV levels in young individuals during the tilt test similar to those of elderly individuals in the supine position.

As observed in this study, although SBP is one of the most important variables accounting for a direct increase in PWV, both at baseline and in orthostatic position (Table 4), no additional increase in SBP was detected during the tilt test compared with its baseline levels (baseline SBP: 130 ± 18 mmHg, SBPp: 128 ± 15 mmHg, NS). Indeed, a decreasing trend was observed in orthostatic position. This finding is in accordance with literature data, which also evidenced lack of a statistically significant increase or even a decreasing trend in SBP in orthostatic position.^14^

Another important aspect is that although this study did not directly assess vasomotor activity, the fact that it found an increase in MAP (baseline MBP: 99 ± 15, MBPp: 103 ± 11, p < 0.05) means that there was probably an increase in peripheral vascular resistance due to reflex sympathetic activation induced by the fall in pulse pressure while standing. Thus, although a variable PWV may be strongly influenced by the MBP, the increased PWV can be attributed to both secondary circulatory disorders due to gravitational stress and increased peripheral vascular resistance, rather than to the effect of high MBP.

Transmission of pulse wave is known to be primarily dependent on arterial elasticity or stiffness coefficient. However, several other factors should be considered. Some of these factors are related to cardiovascular physiology, while others result from specific pathophysiological conditions.^15^ Analyzing the correlation between baseline PWV and PWVp, a direct influence of the baseline pattern of arterial compliance was observed on the response of the great arteries to orthostatic position (Figure 2).

Because no significant increase in SBP was observed in orthostatic position, the increase in PWV may have resulted from circulatory dynamics disorders resulting from gravitational force, in association with structural and geometric characteristics of the aorta. This hypothesis is based on the Moens-Korteweg formula and on the knowledge that PWV depends on vascular radius and thickness, as well as on the vascular elastic module.

Measurement of PWV during the tilt test exposes the arterial segments to gravitational stress in a distinguished form, imitating, to a certain extent, what occurs during active orthostatic position.

In fact, the immediate consequence of orthostasis is that gravity favors a progressive increase in blood pressure in the segments below the cardiac level in orthostatic position.^8,16-18^

**Figure 3:**
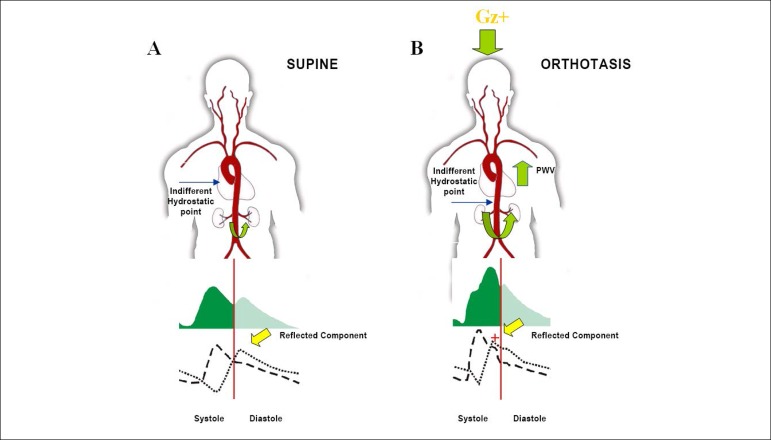
Sketch of the mechanism proposed for pulse wave velocity behavior in a healthy young individual. A – In the supine position, the reflected component occurs during the diastole due to a smaller PWV. B – In orthostatic position, due to the gravitational force, the indifferent hydrostatic point moves to the subdiaphragmatic aorta, which has a smaller radius and a greater elastic module; therefore, PWV increases, leading to an earlier return of the reflected component of the pulse wave, which then occurs with a systolic “pulse summation”. Thus, pulse wave morphology is altered. 321x263 mm (72 x 72 DPI)

The hydrostatic pressure generated by the gravitational force changes the indifferent hydrostatic point, defined as the axial reference in which the venous blood column pressure is not altered by postural reorientation. Such point is located at the right atrial level in supine position and in the infradiaphragmatic aortic territory in orthostatic position. Because of this, an increase in blood flow to the arterial segments with greater elastic module and smaller radius occurs, evidencing the increase in the measured carotid-femoral PWV.^11,16^ This increase in PWV accounts for the early return of the reflected waves from the peripheral sites to the ascending aorta. This wave, reflected earlier (during the ventricular ejection period), adds to the incident wave generated by left ventricular ejection and influences the contour of the pressure and flow waves.^10^ In other words, the earlier return of the reflected component, occurring during the systolic component of the pulse wave, leads to an increase in pulse pressure (pulse summation).^19-20^ This increase, provided by the reflected wave in the initial portion of the arterial pulse wave, may result from a complex, evolutionary, functional, anatomical-humoral adaptation of the vascular system, to maintain an effective cerebral blood flow in response to bipedalism (Figure 3).^21^

The observed increase in PWVp can also be attributed to the emergence of new sites of reflection of the pulse in the peripheral circulation due to a possible increase in peripheral vascular resistance in response to standing.

A phasic response of the central hemodynamics to gravitational stress has been reported in experimental studies with baboons. A later systolic peak resulting from the reflected component of the pulse wave does not occur immediately after taking orthostatic position in these animals. Therefore, the reflected wave appears later in diastole, suggesting a decrease in PWV. Then, the so-called compensatory phase occurs during response to baroreflex.^12,22^

The role played by wave reflection in the circulatory homeostasis in orthostatic position is reinforced by the observation that nitroglycerin, used sublingually for tilt test sensitization, causes peripheral vasodilation, leading to a delay in the reflected component of the pulse wave and consequent reduction in the proximal systolic pressure, culminating in symptoms of low cerebral blood flow in patients with neuromediated syncope.^23-24^

In reality, although a decrease in the coefficient of reflection is observed using nitroglycerin, an increase in aortic stiffness is paradoxically observed. This secondary aortic stiffness was attributed to a possible reflex activation of the sympathetic nervous system.^25^

Analysis of PWV behavior in this series of patients also showed that the increase in PWV was positively correlated with age, showing that, even in the elderly, who have higher baseline PWV levels, an additional increase occurred by taking orthostatic position (r^2^ = 0.357, p < 0.001 in the supine position and r^2^ = 0.406, p < 0.001 in orthostatic position). In reality, this additional increase in PWV in the elderly results from the addition of structural findings with the dynamic postural component.

Another intriguing aspect of this study was the discovery of a negative correlation between HR and PWV in orthostatic position (r = -0.36, p < 0.001). Age was the major variable responsible for HR behavior in orthostatic position (r^2^ = 0.27, p < 0.001).

Aiming at better understanding that finding, the correlation between HR measured during orthostatic stress and baseline PWV was also assessed. A negative correlation between HR and baseline PWV (r = -0.29, p < 0.01) was observed. This result shows a clear association of the baseline pattern of arterial compliance with the level of HR response to orthostatic position.

Initial experimental studies and studies in human beings in the supine position reported a positive correlation between increased HR and increased aortic stiffness.^1,7,26-27^ However, Wilkinson et al.,^14^ in a study assessing PWV behavior and the invasive augmentation index in healthy individuals undergoing atrial stimulation, reported no significant alterations in aortic distensibility due to an increase in HR.^14^

Further to the lack of consensus on the effects of heart rate on PWV, the possible mechanisms contributing to PWV changes with heart rate have yet to be fully elucidated, although many investigators have attributed heart-rate related changes in arterial stiffness to the viscoelasticity of the arterial wall. With high heart rate being an independent prognostic factor of cardiovascular disease and its association with hypertension, the interaction between heart rate and PWV continues to be relevant in assessing cardiovascular risk.^28^

Although a first analysis of these data point to a potential disagreement with our findings, they should be considered as complementary to each other and analyzed within a dynamic context, because they were obtained in very distinct physiological conditions.

Cross-sectional studies have reported that baseline HR does not differ between young and elderly individuals in the supine position.^29-30^ However, HR assessment of healthy individuals in the sitting position has shown that HR decreases with age in both sexes. On the other hand, studies using the tilt test to assess cardiovascular adaptation to orthostatic stress have also reported a significantly lower response of HR in elderly individuals.^16^

The decrease in HR variability due to postural change observed in elderly patients compared with that in young individuals has been attributed to a decrease in the recruitment of the activity of baroreceptors in orthostatic position.^16,29-30^

Considering the dynamic behavior of aortic compliance due to postural change, a greater decrease in systolic volume (SV) in young individuals could lead to a decrease in pulsatile aortic strain, with a subsequent decrease in baroreceptor stimulation and an increase in HR.^31^

Maintenance of systolic volume in elderly individuals has been attributed to lower venous compliance in this group, allowing preservation of cardiac filling volume, and, consequently, of systolic volume.^11^ However, a lower HR associated with age, such as that observed in this study, could also mean an adaptive mechanism of man to bipedalism.

Heart rate is known to affect preload via its effect on diastolic filling time and to modulate the myocardial contractility status, altering the myocardial concentration of Ca^2+^ and Na^+^. As a consequence of increase in myocardial contractility, HR also modulates end-systolic volume, systolic volume and ejection fraction.^11^ Thus, a lower HR could initially allow better cardiac performance in the presence of a stiffer arterial system.

**Figure 4:**
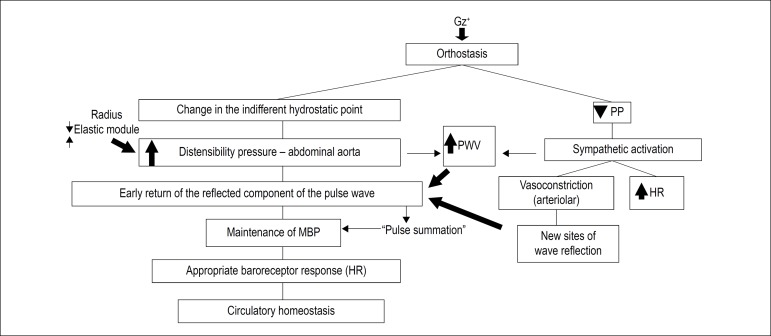
Hypothesis proposed for the role played by the increase in carotid-femoral pulse wave velocity in the maintenance of circulatory homeostasis in response to orthostatic stress. 309x165 mm (72 x 72 DPI). PP: pulse pressure; PWV: pulse wave velocity; MBP: medium blood pressure; HR: heart rate.

Considering all that has been presented so far, the following hypothesis was formulated: the return of the reflected component of the pulse wave is fundamental to the immediate adaptation to orthostatic position, as well as to the adequate adaptation of baroreceptors. Based on data from this study, it is not possible to determine whether this increase is not only due to the dynamic circulatory changes secondary to gravity, but also to sympathetic activation in response to decreased stroke volume and pulse pressure. If the above hypothesis is correct, one may consider the role played by our findings in some very common clinical conditions associated with orthostatic position (Figure 4).^9,32-34^

### Study limitations

This study has some limitations. Assessment of sympathetic response was not performed during the tilt test through venous measurement of catecholamines or recording of neural sympathetic activity through electroneuromyography.^35^ However, although the present study did not directly assess vasomotor activity, an increase in mean arterial pressure (supine: 099 ± 15 mmHg vs. orthostasis: 103 ± 11 mmHg, p < 0.01) allowed us to infer that there has been an increase in peripheral vascular resistance due to the reflex sympathetic activation induced by the drop of pulse pressure in orthostasis.

Another limiting factor was the lack of study of the baroreflex response to orthostatic stress caused by the tilt test, which resulted in lack of data referring to its dysfunction and to the specific location of alterations in its reflex arch. Change in HR was the only response observed, with no other baroreflex measurements, particularly those of systolic volume, although they have been shown to be closely related.^36^

Another possible source of error in PWV measurement lies in determining the arterial segment. Its superficial and noninvasive measurement allows only an estimation of the distance traveled by the pulse wave.

Obtainment of carotid-femoral PWV comprises the analysis of a relatively long arterial segment, which may be extremely tortuous from the three-dimensional point of view. Another factor is that the vessel may be distorted by the direct application of the pressure transducer. This aspect is more evident in deeper vessels.^37^ These observations become potentially more significant when carotid-femoral PWV measurement is considered during the tilt test. The change from supine decubitus to orthostatic position may increase the difficulty in recording femoral pulse, mainly in obese individuals. In some cases, recording femoral pulse is impossible. In this study, we tried to reduce these sources of error by using observers who were very well-trained in measuring PWV in the supine and orthostatic positions.

Although this study does not allow comparison of the influence of sex on arterial behavior in orthostatic position, the inclusion of female individuals in this study may not have interfered with the response of PWV increase to postural stress. Considering the greater arterial distensibility in young females,^38^ a relatively smaller increase could occur in PWV due to postural stress caused by the tilt test. However, this hypothesis cannot be statistically confirmed with the data from this study.^39^

## Conclusion

In conclusion, our study of healthy individuals and in individuals with untreated mild-to-moderate arterial hypertension demonstrates a rapid increase in PWV during the tilt test and its return to baseline levels when resuming to supine position. We found strong indications that these results may enable a better comprehension of the response of the great arteries to gravitational stress. These results may also elucidate this behavior in the long run, in the presence of inherent degenerative disorders, such as arterial hypertension and aging, and predetermined genetic markers.
